# Mitochondrial Genomes of the Genus *Claassenia* (Plecoptera: Perlidae) and Phylogenetic Assignment to Subfamily Perlinae

**DOI:** 10.3390/genes12121986

**Published:** 2021-12-14

**Authors:** Yanan Xiang, Mengyuan Zhao, Qingbo Huo, Yuzhou Du

**Affiliations:** School of Horticulture and Plant Protection & Institute of Applied Entomology, Yangzhou University, Yangzhou 225009, China; xynxiang@163.com (Y.X.); zhaomy1012@163.com (M.Z.); jw30th@163.com (Q.H.)

**Keywords:** mitochondrial genome, *Claassenia*, Plecoptera, phylogeny

## Abstract

Mitochondrial genomes of three stoneflies, e.g., *Claassenia magna* Wu, 1948, *Claassenia* sp. 2 and *Claassenia xucheni* Chen, 2019 were sequenced in this study with 15,774, 15,777 and 15,746 bp in length, respectively. Each mitogenome contained 37 genes including 22 tRNAs, two ribosomal RNAs, 13 protein-coding genes (PCGs), and a noncoding control region (CR). In general, standard ATN start and TAN termination codons were evident in the PCGs. Although the dihydrouridine arm was absent in *trnSer*, the remaining 21 tRNAs displayed the characteristic cloverleaf secondary structure. Stem-loop structures were identified in the CRs of all three mitogenomes, but tandem repeats were only apparent in *Claassenia xucheni*. The mitogenomes of three *Claassenia* species were analyzed and compared with mitogenomes in 21 other stoneflies from the Perlidae and three Euholognatha species (*Rhopalopsole bulbifera*, *Capnia zijinshana* and *Amphinemura longispina*) as outgroups. Phylogenetic analyses using maximum likelihood and Bayesian inference. Phylogenetic analysis supported that *Claassenia* was recovered as the sister group of other Perlinae and *Claassenia*+Perlinae emerged from the paraphyletic Acroneuriinae. The final results supported that *Claassenia* was classified into subfamily Perlinae and proposed *Claassenia* represent a transitional group of the subfamilies Acroneuriinae and Perlinae. This study provided new molecular evidence for exploring the debatable taxonomic position of the genus *Claassenia* in Perlidae.

## 1. Introduction

Analysis of mitochondrial genomes is widely used in evolutionary biology, population genetics, taxonomy, and phylogenetics [1] and has been used to resolve phylogeny in many insect species [2,3,4,5,6,7,8,9,10,11,12,13]. A typical mitogenome consists of a noncoding sequence called the control region (CR) and two rRNAs, 22 tRNAs, and 13 protein-coding genes (PCGs) [14,15]. Both gene organization and nucleotide composition of mitogenomes have evolutionary and phylogenetic significance for insects [16].

Plecoptera (order: Stoneflies) species are hemimetabolous insects, a small order of insects. The Claasseniini is a tribe in Perlidae that includes only the genus *Claassenia* containing 13 species worldwide [17,18,19]. The genus *Claassenia* Wu, 1934, was proposed as a replacement for *Adelungia* Klapálek, 1914 [20,21]. *Claassenia* was placed in the subfamily Acroneuriinae because of a circular hammer presented at the median near the hind margin of sternum 9, which is an important morphological characteristic of the subfamily Acroneuriinae [20]. Stark and Gaufin moved it to the subfamily Perlinae [22], because of the cleft in tergum 10 that is a synapomorphic characteristic of the subfamily Perlinae. So it is uncertain and debatable to determine the taxonomic status of *Claassenia* by the morphological characteristics.

Therefore, it is necessary to use mitogenomic evidence to explore the phylogenetic relationship of *Claassenia* in Perlidae. Up to now, although 78 species of Plecoptera have been sequenced and listed in the NCBI GeneBank, there was only one mitogenome in *Claassenia* has been previously reported [23,24]. Chen et al. (2019) proposed that Acroneuriinae + Perlinae was a sister clade to *Claassenia*, based on 10 Perlidae and two Capniidae species as outgroups [23]. Based on 16 Perlidae species and two Capniidae species as outgroups, Wang et al. (2020) supported that *Claassenia* was a sister taxon to Acroneuriinae + Perlinae [25]. However, Wang et al. (2020) reported another phylogenetic analysis with 16 species in Perlidae and two species in Taeniopterygidae as outgroups subsequently, this analysis showed that Perlinae and *Claassenia* were clustered in a clade and emerged from paraphyletic Acroneuriinae [26]. The relationship of *Claassenia* with Acroneuriinae and Perlinae is still unstable because of the limited mitogenomes. Here, we sequenced three mitogenomes of *Claassenia*, downloaded almost all the mitogenomes of Perlidae species (including one *Claassenia* species, nine Acroneuriinae species, 11 Perlinae species) and three Euholognatha species (as outgroups) from GeneBank, these data were used to construct phylogenetic trees based on 13 PCGs to deduce the phylogenetic relationship of the genus *Claassenia* in Perlidae.

## 2. Materials and Methods

### 2.1. Sample Preparation and DNA Extraction

This study was conducted without harming protected or endangered species and all research activities were authorized. *C. magna* was collected from Fujian Province, China in May 2021, *Claassenia* sp. 2 was collected from Tibet, China in July 2020, and *C. xucheni* was collected from Shaanxi Province, China in May 2021; all specimens were preserved in 100% ethanol and stored at −20 °C. Genomic DNA was extracted from the legs of specimens with the Column mtDNAout Kit (Axygen Biotechnology, Hangzhou, China) as recommended by the manufacturer and stored at −20 °C until used for PCR.

### 2.2. PCR Amplification and Sequencing

Mitochondrial genome was amplified using LA-PCR and continuous specific PCR amplification as the following conditions: perform initial denaturation at 93 °C for 2 min, and then perform 40 cycles at 92 °C for 10 s; annealing at 54 °C for 30 s; and stretching at 68 °C (20 cycles) for 8 min Elongation rate, which increases by 20 s/cycle in the last 20 cycles; the final extension is 10 min at 68 °C. PCR products were purified with Axygen DNA Gel Extraction Kit (Axygen Biotechnology, Hangzhou, China) [16], and quality control was subsequently carried out on the purified DNA samples. The quality of DNA was assessed using qubit3.0 and 1% agarose gel electrophoresis.

High qualified DNA samples were applied to 500-bp paired-end library construction using the NEBNext Ultra DNA Library Prep Kit for Illumina sequencing. Sequencing was carried out on the Illumina NovaSeq 6000 platform (BIOZERON Co., Ltd., Shanghai, China). De novo assembly with GetOrganelle v1.6.4 referencing mitogenome of closely related species produced contigs of mitogenome. A number of potential mitochondrion reads were extracted from the pool of Illumina reads using BLAST searches against mitogenomes of related species and the GetOrganelle results. The mitochondrion Illumina reads were obtained to perform complete mitogenome de novo assembly using the SPAdes-3.13.1 package. The GetOrganelle assembly contig was optimized by the scaffolds from SPAdes-3.13.0 result. Finally, the assembled sequences were reordered and oriented according to the reference mitogenome, thus generating the final assembled mitochondrion genomic sequence (BIOZERON Co., Ltd., Shanghai, China).

### 2.3. Mitogenome Assembly and Annotation

The assembly of mitogenomes was conducted with CodonCode Aligner (http://www.codoncode.com/aligner/, accessed on 7 November 2021). Mitogenomes from other members of the Plecoptera were used to identify genes encoding PCGs and rRNAs, and ORFs were delimited by ORF finder (https://www.ncbi.nlm.nih.gov/orffinder/, 7 November 2021). The circular mitogenome maps were drawn using the CGview tool (http:// stothard.afns.ualberta.ca/cgview_server/, 7 November 2021) [27]. The mitochondrion genes were annotated using the online MITOS tool [28], and the ARWEN program was used to predict tRNA secondary structure (http://mbio-serv2.mbioekol.lu.se/ARWEN/, 7 November 2021). MEGA v. 6.0 [29] was used to obtain and analyze nucleotide composition. Composition skew analysis was performed using the formulas AT-skew = [A − T]/[A + T] and GC-skew = [G − C]/[G + C] [30]. The stem-loops (SL) structure was predicted by DNAMAN v. 6.0.3 using the complementary function of primers. Tandem Repeats Finder (http://tandem.bu.edu/trf/trf.advanced.submit.html, 7 November 2021) was used to analyze the tandem repeats in the putative control region (CR). Mitogenome sequences of *C. magna, Claassenia* sp. 2 and *C. xucheni* were deposited in GenBank as OK012602, OK021652 and OK021653, respectively (Table 1).

### 2.4. Phylogenetic Analysis

Twenty-seven Plecoptera mitogenomes were analyzed, including nine species of the subfamily Acroneuriinae, 11 from the Perlinae and four from the genus *Claassenia* (*Claassenia* sp. 1 was downloaded from GenBank accession no. MN419914). Three Euholognatha species (*Rhopalopsole bulbifera*, *Capnia zijinshana* and *Amphinemura longispina*) were used as outgroup species (Table 1). The 13 PCGs in the 27 mitogenomes were assembled using SequenceMatrix v. 1.7.8 [31] and MAFFT [32]; stop codons were not included. Sequence alignment and file format conversion using MEGA v. 6.0 [29]. DAMBE v. 5.2 (http://dambe.bio.uottawa.ca/DAMBE/dambe.aspx, 7 November 2021) was used to determine nucleotide saturation prior to the phylogenetic tree construction. The best nucleotide substitution model was determined with MEGA v. 6.0 using the Bayesian Information Criterion (BIC) and the GTR+G+I model was predetermined for analyses. Using MrBayes v. 3.1.2 (http:// morphbank.ebc.uu.SE/mrbayes/, 7 November 2021) with 20 million generations to conduct Bayesian inference analysis; sampling every 100 generations with four chains (three hot and one cold), and a burn-in of 25% trees [13,33]. IQ-Tree v. 1.6.12 (http://www.iqtree.org/, 7 November 2021) [34,35] was used for maximum likelihood with 1000 Ultrafast bootstrap approximations. The phylogenetic trees were annotated with FigTree v. 1.4.2 (http://tree.bio.ed.ac.uk/software/figtree/, 7 November 2021).

## 3. Results

### 3.1. Mitogenome and Base Composition

**Figure 1:**
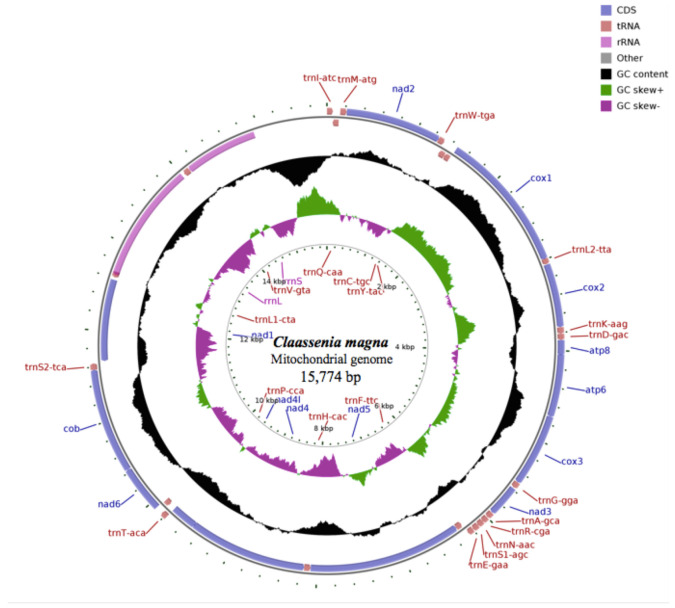
Mitochondrial maps of *C. magna*. Genes outside the map are transcribed clockwise, whereas genes inside the map are transcribed counterclockwise. The interior circles show GC content and the GC skew, and these are plotted as the deviation from the average value of the entire sequence.

Mitogenomes of *C. magna* and *Claassenia* sp. 2 and *C. xucheni* were circular DNA molecules consisting of 15,774, 15,777 and 15,746 bp, respectively, which are ranges consistent with mitogenomes in other stoneflies [36]. The three mitogenomes encoded a large noncoding control region and two rRNAs, 22 tRNAs and 13 PCGs. Twenty-three genes (14 tRNAs and nine PCGs) were located on the majority J-strand and 14 genes (two rRNAs, eight tRNAs, and four PCGs) were on the minority N-strand (Figure 1, Tables S1–S3). The order of genes in the three *Claassenia* mitogenomes was conserved with other stoneflies and identical to the ancestral mitogenome of *Drosophila yakuba* [37]. In *C. magna*, 70 overlapping nucleotides were located in 11 pairs of neighboring genes; whereas 89 overlapping nucleotides were found in 14 pairs of neighboring genes in *Claassenia* sp.2 and *C. xucheni* contained 69 nucleotide overlaps with 13 gene pairs.

In *C. magna, Claassenia* sp. 2 and *C. xucheni*, the A+T content was as follows: 61.46%, 65.81% and 62.89% (whole mitogenomes); 59.40%, 64.12% and 60.62% (PCGs); 66.98%, 68.70% and 67.40% (tRNAs); 64.15%, 69.04% and 67.06% (rRNAs); and 71.51%, 74.45% and 73.95% (CRs), respectively (Table 2). In *C. magna*, the highest and lowest A+T content was 76.12% for *trnAsp* (D) and *trnthr* (T) and 53.72% for *cox1.* In *Claassenia* sp. 2, the highest and lowest A+T content was 78.26% for *trnAsp* (D) and 55.55% for *trnVal* (V); whereas *trnthr* (T) was 78.46% and *cox3* was 55.09% in *C. xucheni*, respectively (Tables S1–S3). The A+T contents of whole mitogenomes, PCGs, tRNAs, rRNAs and CRs genes in *Claassenia* sp. 2 were all the highest. This phenomenon may be related to its distribution in the Qinghai Tibet Plateau where the environment was harsh. In view of this phenomenon, it is necessary to collect more specimens from different environments and extract more molecular data for more accurate exploration.

### 3.2. Protein-Coding Genes

The 13 PCGs of the three *Claassenia* mitogenomes were similar in size and A + T content (Table 2). The majority of the PCGs in all three mitogenomes initiated with the standard start codon ATN (ATT, ATC, ATA and ATG); however, *cox1* in *C. magna* started with CAA and *nad1, nad4* and *nad5* used GTG as a start codon. In *Claassenia* sp. 2, *nad1* and *nad5* initiated with TTG and GTG, respectively; whereas *nad1* started with GTG in *C. xucheni* (Tables S1–S3). Most PCGs had standard stop codons (TAA or TAG); however, *cox2* and *nad5* in both *C. magna* and *Claassenia* sp. 2 and *cox2*, *nad4, nad5* in *C. xucheni* contained a truncated termination codon (‘T’), which is likely completed by post-transcriptional polyadenylation [38]. Some PCG genes used nonstandard start codons or stop codons, these phenomena are common in Plecoptera [6,7,8].

**Figure 2:**
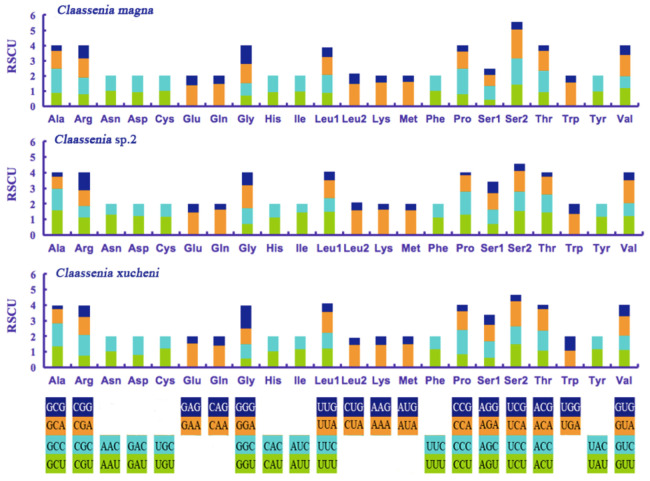
Relative synonymous codon usage (RSCU) in mitogenomes of the three *Claassenia* species.

The relative synonymous codon usage (RSCU) values of the three mitogenomes were calculated. In *C. magna*, GCC (*Ala*), GAA (*Glu*), CAA (*Gln*), CTA (*Leu2*), AAA (*Lys*), ATA (*Met*) and TCA (*Ser2*) were relatively high, whereas GCG (Ala) was used the least (Figure 2). In *Claassenia* sp. 2, GAA (*Glu*), CAA (*Gln*), CTA (*Leu2*), AAA (*Lys*) and ATA (*Met*) were frequently used, whereas CCG (*Pro*) was seldom utilized. In *C. xucheni*, GCC (*Ala*), GAA (*Glu*), CAA (*Gln*), CTA (*Leu2*), AAA (*Lys*), ATA (*Met*) and TCA (*Ser2*) were used frequently, whereas GCG (*Ala*) was seldom used (Figure 2). GAA (*Glu*), CAA (*Gln*), CTA (*Leu2*), AAA (*Lys*), ATA (*Met*) were the most frequently commonly used of the three species, this was a little different from other Plecoptera species [16,36], so we inferred that this may be endemic to the genus *Claassenia*. However, more molecular data are needed to provide evidence.

### 3.3. Transfer RNA Genes

**Figure 3:**
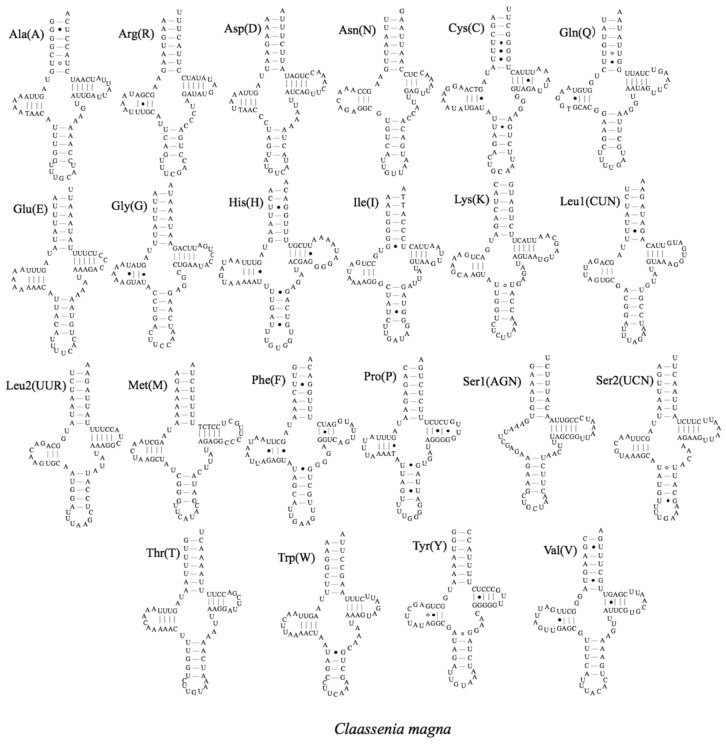
Predicted secondary structures of tRNAs in *C. magna*. The tRNAs are labelled with abbreviations of their corresponding amino acids.

The typical set of 22 tRNA genes was predicted from the three mitogenomes. The lengths of *C. magna, Claassenia* sp. 2 and *C. xucheni* tRNA genes were 1493 bp, 1492 bp and 1491 bp, and the A+T content of tRNA genes was 66.98%, 68.70% and 67.40%, respectively (Table 2). Most tRNAs had a typical cloverleaf secondary structure (Figure 3, Figures S3 and S4); however, in *trnSer* (AGN), the dihydrouridine (DHU) arm was missing in the three species, which is common in mammals and some insects [39]. The anticodons of the 22 tRNAs in the three *Claassenia* species were identical to other stoneflies. The tRNAs contained mismatched base pairs, and most of these were G–U pairs (Figure 3, Figures S3 and S4).

### 3.4. Ribosomal RNA Genes

There were two rRNAs predicted in each mitogenome, and their total length and A+T content were basically similar (Table 2). Like other Plecoptera species, the two rRNA genes mapped in the conserved location between *trnLeu* (CUN) and the control region (Figure 1, Figures S1 and S2). The *rrnL* gene was 1,365 bp with an A+T content of 65.27% in *C. magna*, 1392 bp with an A+T content of 69.68% in *Claassenia* sp. 2 and 1371 bp with an A+T content of 67.98% in *C. xucheni.* Meanwhile, the small ribosomal RNA (*rrnS*) gene was 836 bp with an A+T content of 62.32% in *C. magna*, 830 bp with an A+T content of 67.95% in *Claassenia* sp. 2 and 833 bp with an A+T content of 65.54 % in *C. xucheni* (Tables S1–S3).

### 3.5. The Non-Coding Control Region

The mitogenome control regions are highly variable with respect to length and nucleotide composition. The A + T content of CR in *C. magna, Claassenia* sp. 2 and *C. xucheni* was 71.51%, 74.45% and 73.95%, respectively (Table 2), the differences were not obvious and within the scope of all sequenced stoneflies [36]. The CR in *C. magna, Claassenia* sp. 2 and *C. xucheni* mapped between *rrnS* and *trnIle*, which is a relatively conserved position in stoneflies (Figure 1, Figures S1 and S2).

**Figure 4:**
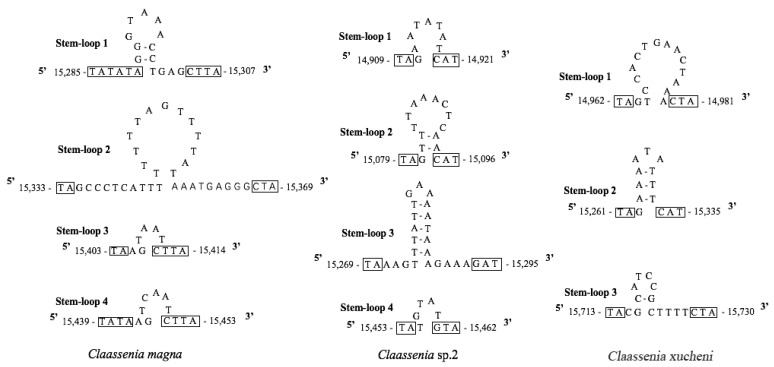
The potential stem-loop structures in the control region of three *Claassenia* species. The bilateral nucleotide motifs of each stem-loop structure [(TA)_n_, CAT, C(T)_n_A, GTA] are indicated by rectangles.

The CR of *C. magna* contained four stem-loop (SL) structures (15,285–15,307 bp; 15,333–15,369 bp; 15,403–15,414; 15,439–15,453), and the CR of *Claassenia* sp. 2 contained four SL structures (14,909–14,921 bp; 15,079–15,096 bp; 15,269–15,295 bp; 15,453–15,462 bp). The CR of *C. xucheni* contained three SL structures (14,962–14,981 bp; 15,261–15,335 bp; 15,713–15,730 bp) (Figure 4). The stem-loops structure was a single vertical root, there were (TA) n structure on the left and G (A) nT, GT (A)n, GCAT, CAT, or C (T) nA structures on the right. These SL structures were considered to be related to the initiation of mitogenome replication and transcription 40. There was only one tandem repeat between 15,378–15,421 bp in *C. xucheni*, the absence of tandem repeats in the other two species maybe can be accounted for insertion and deletion events, and differences in variable domain length. However, their structural patterns, variations, and functions are still indistinct, although great quantity stonefly CRs data have been provided [16].

### 3.6. Phylogenetic Analyses

**Figure 5:**
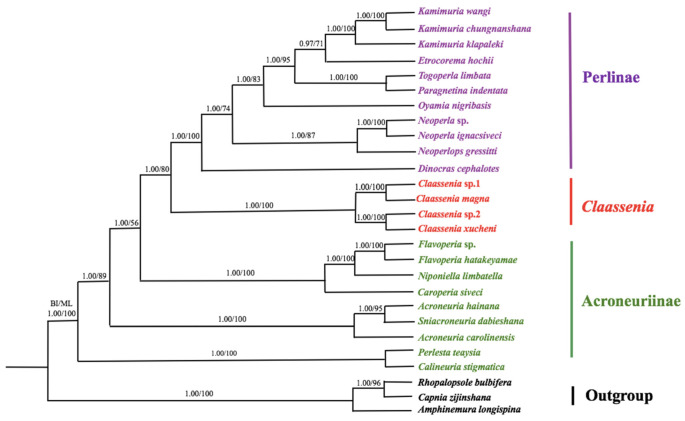
Phylogenetic tree based on mitogenomes of 27 stoneflies by using Bayesian inference (BI) and Maximum Likelihood (ML). Numbers at nodes represent posterior probabilities (left) and bootstrap values (right). Subfamily names and the genus *Claassenia* are marked to the right of each species. *R. bulbifera*, *C. zijinshana* and *A. longispina* served as outgroup species.

The phylogenetic analyses were constructed by using concatenated sequences of 13 PCGs from 27 stoneflies. These mitogenomes included nine species from subfamily Acroneuriinae, 11 from Perlinae, one *Claassenia* sp. 1 (GenBank accession no. MN419914), and *Claassenia magna*, *Claassenia* sp. 2, and *Claassenia xucheni* which were sequenced by this study. Three Euholognatha species (*Rhopalopsole bulbifera*, *Capnia zijinshana* and *Amphinemura longispina*) were included as outgroups (Table 1). Tree structures were similar for dendrograms generated by Bayesian inference (BI) and maximum likelihood (ML) analyses, and species grouped with high support values (Figure 5).

In the two analyses, *Claassenia* was recovered as the sister group of other Perlinae, they were grouped together and emerged from a paraphyletic Acroneuriinae. So our results supported that the genus *Claassenia* was classified into subfamily Perlinae at present based on molecular data, this corresponds with the current taxonomic position of *Claassenia* based on morphological characteristics. In addition, *Claassenia* was monophyletic in the phylogenetic tree, we proposed that the genus *Claassenia* was a transitional group of the two subfamilies Acroneuriinae and Perlinae.

However, in the phylogenetic tree, the Acroneuriini species *Calineuria stigmatica* grouped with the Kiotinini species *Perlesta teaysia* (Figure 5). They belong to different tribes, but both of them are distributed in North America and eastern Asia [17]. Maybe it can be explained by animal geography. However, due to the limitations of mitochondrial genes, their relationship is still unclear, more gene sequencing is necessary to explore this problem.

## 4. Discussion

In this study, we sequenced three *Claassenia* mitogenomes and downloaded all the data of Perlidae species (except one *Togoperla* sp.) from GenBank to present phylogenetic analyses. The results showed that *Claassenia* was monophyletic and grouped with Perlinae. Acroneuriinae was paraphyletic and Perlinae + *Claassenia* emerged from Acroneuriinae. The final relationship was listed as follows: (Perlinae + *Claassenia*) + Acroneuriinae (Figure 5). However, Chen et al. (2019) resulted that Acroneuriinae + Perlinae was a sister clade to *Claassenia*, based on 10 Perlidae and two Capniidae species as outgroups [23]. The difference between our results may come from his use of limited mitogenomic data. Based on 16 Perlidae species and two Capniidae species as outgroups, Wang et al. (2020) supported that *Claassenia* was a sister taxon to Acroneuriinae + Perlinae [25], which was consistent with Chen (2019). Subsequently, Wang et al. (2020) reported another phylogenetic analysis with 16 species in Perlidae and two species in Taeniopterygidae as outgroups. This analysis showed that Perlinae and *Claassenia* were clustered in a clade and emerged from paraphyletic Acroneuriinae [26], this result was consistent with us. The two inconsistent results of Wang may be caused by the different choices of outgroups. The selection of outgroups is very crucial in phylogenetic analysis.

To sum up, the prior assignments of genus *Claassenia* in the Perlidae were inconsistent based on mitogenomes and uncertain based on morphology due to *Claassenia* having the identical derived characters of the subfamily Perlinae and the important morphological characteristic of the subfamily Acroneuriinae. Besides, Duran et al. (2020) found that mitogenomes were inconsistent with genomic divergences and species-level taxonomy, and consequently, taxon identifications based on mitogenomes (e.g., DNA barcodes) may be misleading [41]. So more molecular evidence other than mitogenomes can be considered for reference and morphological characteristics of more eggs, nymphs and adults of the genus are needed to deduce its relationship with Perlinae and Acroeuriinae.

## Figures and Tables

**Table 1:** List of species analyzed in this study.

Order	Subfamily	Species	GenBank Accession No.
Plecoptera	Perlinae	*Dinocras cephalotes*	KF484757
		*Neoperla* sp. FS-2017	KX091859
		*Neoperla ignacsiveci*	KX091858
		*Neoperlops gressitti*	MN400756
		*Oyamia nigribasis*	MN548290
		*Kamimuria wangi*	KC894944
		*Kamimuria chungnanshana*	KT186102
		*Kamimuria klapaleki*	MN400755
		*Paragnetina indentata*	MN627431
		*Togoperla limbata*	MN969990
		*Etrocorema hochii*	MK905888
		*Claassenia* sp. YW-2019	MN419914
		*Claassenia magna*	OK012602
		*Claassenia* sp. 2	OK021652
		*Claassenia xucheni*	OK021653
	Acroneuriinae	*Sniacroneuria dabieshana*	MK492253
		*Acroneuria hainana*	KM199685
		*Acroneuria carolinensis*	MN969989
		*Perlesta teaysia*	MN627432
		*Calineuria stigmatica*	MG677941
		*Flavoperla* sp. YZD-2020	MK905206
		*Flavoperia hatakeyamae*	MN821010
		*Niponiella limbatella*	MK686067
		*Caroperia siveci*	MG677942
	Leuctrinae	*Rhopalopsole bulbifera*	MK111419
	Nemouroidea	*Capnia zijinshana*	KX094942
	Amphinemurinae	*Amphinemura longispina*	MH085446

**Table 2:** A+T content in different regions of *C. magna*, *Claassenia* sp. 2 and *C. xucheni* mitogenome.

Species	Whole Genome		PCGs		tRNAs		rRNAs		Control Region	
	Size(bp)	A+T (%)	Size (bp)	A+T (%)	Size (bp)	A+T (%)	Size (bp)	A+T (%)	Size (bp)	A+T (%)
*Claassenia magna*	15,774	61.46	11,177	59.40	1493	66.98	2201	64.15	832	71.51
*Claassenia* sp. 2	15,777	65.81	11,232	64.12	1492	68.70	2222	69.04	869	74.45
*Claassenia* *xucheni*	15,746	62.89	11,139	60.62	1491	67.40	2204	67.06	832	73.95

## Data Availability

Data will be available on reasonable request.

## Supplementary Materials

The following are available online at https://www.mdpi.com/article/10.3390/genes12121986/s1, Table S1: Organization of the *C. magna* mitogenome; Table S2: Organization of the *Claassenia* sp. 2 mitogenome; Table S3: Organization of the *C. xucheni* mitogenome; Figure S1: Mitochondrial maps of Claassenia sp. 2. Genes outside the map are transcribed clockwise, whereas genes inside the map are transcribed counterclockwise. The interior circles show GC content and the GC skew, and these are plotted as the deviation from the average value of the entire sequence; Figure S2: Mitochondrial maps of *C. xucheni*. Genes outside the map are transcribed clockwise, whereas genes inside the map are transcribed counterclockwise. The interior circles show GC content and the GC skew, and these are plotted as the deviation from the average value of the entire sequence; Figure S3: Predicted secondary structures of tRNAs in *claassenia* sp.2. The tRNAs are labelled with abbreviations of their corresponding amino acids; Figure S4: Predicted secondary structures of tRNAs in *C. xucheni*. The tRNAs are labelled with abbreviations of their corresponding amino acids.
