# Supplementary material for: Mitochondrial Genomes of the Genus Claassenia (Plecoptera: Perlidae) and Phylogenetic Assignment to Subfamily Perlinae
Source: Genes (Basel). 2021 Dec 14;12(12):1986. doi: 10.3390/genes12121986 (PMC8701382; doi:10.3390/genes12121986)
Supplement: Supplementary file 1 [file genes-12-01986-s001.zip › genes-1489249-supplementary.pdf]

Table S1. Organization of the *C. magna* mitogenome.

Gene	Position (bp)	Size (bp)	Direction	Intergenic Nucleotides (IGN)	Anti- or Start/Stop Codons	A+T %
trnIle (I)	1–65	65	Forward	0	AAT	63.08
trnGln (Q)	63–131	69	Reverse	−3	TAT	65.22
trnMet (M)	144–213	70	Forward	12	AGA	58.57
nad2	214–1248	1035	Forward	0	ATG/TAA	58.72
trnTrp (W)	1247–1314	68	Forward	−2	AAG	70.59
trnCys (C)	1319–1388	70	Reverse	4	AGC	65.72
trnTyr (Y)	1389–1454	66	Reverse	0	GGT	54.55
cox1	1464–2996	1533	Forward	9	CAA/TAA	53.72
trnLeu2 (UUR)	2992–3055	64	Forward	−5	TCT	67.19
cox2	3059–3746	688	Forward	3	ATG/T-	57.21
trnLys (K)	3747–3816	70	Forward	0	CAT	64.29
trnAsp (D)	3817–3883	67	Forward	0	AAA	76.12
atp8	3884–4045	162	Forward	0	ATG/TAA	66.67
atp6	4039–4716	678	Forward	−7	ATG/TAA	58.96
cox3	4716–5504	789	Forward	−1	ATG/TAA	55.73
trnGly (G)	5506–5574	69	Forward	1	ATT	71.01
nad3	5584–5928	345	Forward	9	ATA/TAA	58.19
trnAla (A)	5933–5999	67	Forward	4	GGG	68.66
trnArg (R)	6004–6068	65	Forward	4	AAG	66.16
trnAsn (N)	6068–6132	65	Forward	−1	TTA	63.07
trnSer1 (AGN)	6133–6199	67	Forward	0	GAA	64.18
trnGlu (E)	6202–6266	65	Forward	2	ATT	83.08
trnPhe (F)	6267–6336	70	Reverse	0	GTT	62.86
nad5	6337–8071	1735	Reverse	0	GTG/T-	62.57
trnHis (H)	8072–8145	74	Reverse	0	ACT	67.57
nad4	8145–9485	1341	Reverse	−1	GTG/TAA	62.7
nad4l	9479–9775	297	Reverse	−7	ATG/TAA	68.37
trnThr (T)	9778–9844	67	Forward	2	GTT	76.12
trnPro (P)	9846–9911	66	Reverse	1	CAG	66.67
nad6	9925–10437	513	Forward	13	ATT/TAA	59.88
Cytb	10437–11573	1137	Forward	−1	ATG/TAG	55.38
trnSer2 (UCN)	11572–11641	70	Forward	−2	AGT	75.71
nad1	11658–12581	924	Reverse	16	GTG/TAG	64.17
trnLeu1 (CUN)	12610–12676	67	Reverse	28	TCT	62.69
rrnL	12637–14001	1365	Reverse	−40		65.27
trnVal (V)	14034–14105	72	Reverse	32	CGA	61.11
rrnS	14107–14942	836	Reverse	1		62.32
CR	14943–15774	832		0		71.51

Table S2. Organization of the *Claassenia* sp. 2 mitogenome.

Gene	Position (bp)	Size (bp)	Direction	Intergenic Nucleotides (IGN)	Anti- or Start/Stop Codons	A+T %
trnIle (I)	1–66	66	Forward	0	AAT	68.18

trnGln (Q)	64–132	69	Reverse	−3	TAT	71.01
trnMet (M)	143–210	68	Forward	10	AGA	60.29
nad2	214–1245	1032	Forward	3	ATC/TAA	65.89
trnTrp (W)	1244–1310	67	Forward	−2	AAG	73.14
trnCys (C)	1303–1370	68	Reverse	−8	AGC	63.23
trnTyr (Y)	1371–1437	67	Reverse	0	GGT	59.71
cox1	1430–2974	1545	Forward	−8	ATT/TAA	60.51
trnLeu2 (UUR)	2970–3033	64	Forward	−5	TCT	64.06
cox2	3038–3725	688	Forward	4	ATG/T-	60.41
trnLys (K)	3726–3795	70	Forward	0	CAT	65.72
trnAsp (D)	3796–3864	69	Forward	0	AAA	78.26
atp8	3865–4026	162	Forward	0	ATC/TAA	66.04
atp6	4020–4697	678	Forward	−7	ATG/TAA	61.78
cox3	4697–5485	789	Forward	−1	ATG/TAA	59.16
trnGly (G)	5485–5551	67	Forward	−1	ATC	74.63
nad3	5552–5905	354	Forward	0	ATG/TAA	61.54
trnAla (A)	5910–5976	67	Forward	4	GGG	73.14
trnArg (R)	5981–6046	66	Forward	4	AAG	65.15
trnAsn (N)	6046–6111	66	Forward	−1	TTA	65.15
trnSer1 (AGN)	6112–6178	67	Forward	0	GAA	64.18
trnGlu (E)	6181–6245	65	Forward	2	ATT	87.7
trnPhe (F)	6245–6316	72	Reverse	−1	ACT	68.05
nad5	6317–8051	1735	Reverse	0	GTG/T-	67.64
trnHis (H)	8052–8123	72	Reverse	0	ACT	69.45
nad4	8124–9462	1339	Reverse	0	ATG/T-	67.34
nad4l	9456–9752	297	Reverse	−7	ATG/TAA	72.11
trnThr (T)	9755–9821	67	Forward	2	GTT	77.61
trnPro (P)	9823–9887	65	Reverse	1	CAG	66.15
nad6	9889–10413	525	Forward	1	ATT/TAA	63.41
Cytb	10413–11549	1137	Forward	−1	ATG/TAG	61.02
trnSer2 (UCN)	11548–11618	71	Forward	−2	AGT	74.65
nad1	11636–12586	951	Reverse	17	TTG	66.77
trnLeu1 (CUN)	12588–12654	67	Reverse	1	TCT	67.17
rrnL	12613–14004	1392	Reverse	–42		69.68
trnVal (V)	14006–14077	72	Reverse	1	CAA	55.55
rrnS	14079–14908	830	Reverse	1		67.95
CR	14909–15777	869		0		74.45

Table S3. Organization of the *C. xucheni* mitogenome.

Gene	Position (bp)	Size (bp)	Direction	Intergenic Nucleotides (IGN)	Anti- or Start/Stop Codons	A+T %
trnIle (I)	1–66	66	Forward	0	AAT	60.6
trnGln (Q)	64–132	69	Reverse	−3	TAT	69.56
trnMet (M)	144–213	70	Forward	11	AGA	61.43
nad2	217–1248	1032	Forward	3	ATC/TAA	60.35
trnTrp (W)	1247–1313	67	Forward	−2	AAG	73.14
trnCys (C)	1306–1373	68	Reverse	−8	AGC	60.29
trnTyr (Y)	1374–1438	65	Reverse	0	GGT	63.08
cox1	1431–2975	1545	Forward	−8	ATT/TAA	56.49
trnLeu2 (UUR)	2971–3034	64	Forward	−5	TCT	64.06
cox2	3039–3726	688	Forward	4	ATG/T-	56.63

trnLys (K)	3727–3796	70	Forward	0	CAT	65.72
trnAsp (D)	3797–3864	68	Forward	0	AAA	75
atp8	3865–4026	162	Forward	0	ATT/TAA	64.78
atp6	4020–4697	678	Forward	−7	ATG/TAA	59.26
cox3	4697–5485	789	Forward	−1	ATG/TAA	55.09
trnGly (G)	5485–5551	67	Forward	−1	ACC	68.66
nad3	5555–5905	351	Forward	3	ATA/TAA	62.07
trnAla (A)	5910–5976	67	Forward	4	GGG	70.15
trnArg (R)	5982–6048	67	Forward	5	AAG	64.18
trnAsn (N)	6048–6113	66	Forward	−1	TTA	65.15
trnSer1 (AGN)	6114–6180	67	Forward	0	GAA	64.18
trnGlu (E)	6185–6250	66	Forward	4	ATT	83.33
trnPhe (F)	6250–6321	72	Reverse	−1	ACT	65.27
nad5	6322–8020	1699	Reverse	0	ATT/T-	64.07
trnHis (H)	8057–8127	71	Reverse	36	ACT	70.43
nad4	8128–9466	1339	Reverse	0	ATG/T-	65.85
nad4l	9460–9756	297	Reverse	−7	ATG/TAA	68.71
trnThr (T)	9759–9823	65	Forward	2	GTT	78.46
trnPro (P)	9825–9890	66	Reverse	1	CAG	68.18
nad6	9904–10416	513	Forward	13	ATC/TAA	57.65
Cytb	10428–11552	1125	Forward	11	ATA/TAG	57.21
trnSer2 (UCN)	11551–11621	71	Forward	−2	AGT	73.24
nad1	11640–12560	921	Reverse	18	GTG/TAG	63.29
trnLeu1 (CUN)	12592–12658	67	Reverse	31	TCT	62.69
rrnL	12636–14006	1371	Reverse	−23		67.98
trnVal (V)	14008–14079	72	Reverse	1	CAA	56.94
rrnS	14081–14913	833	Reverse	1		65.54
CR	14914–15746	832		0		73.95

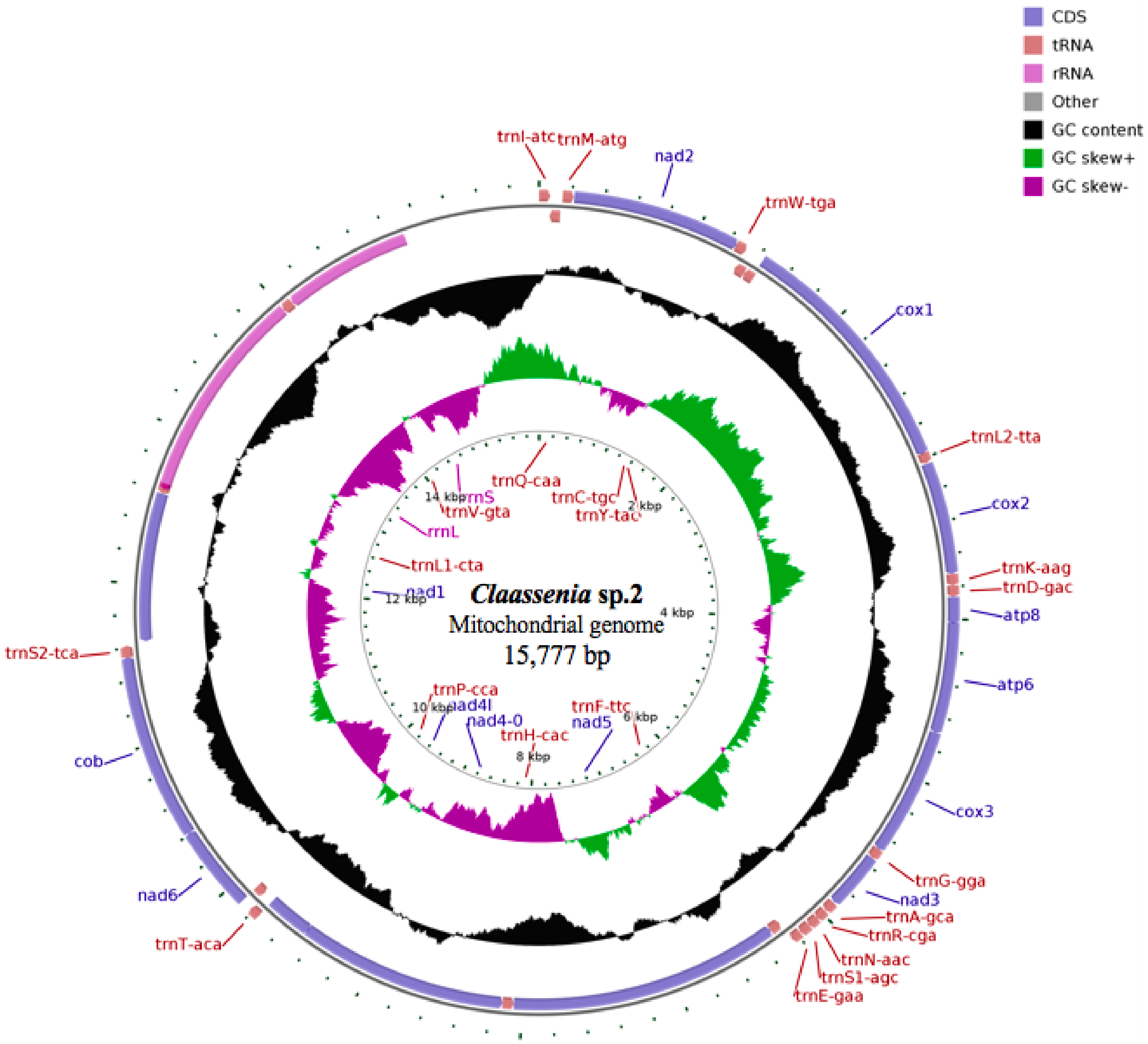

Figure S1. Mitochondrial maps of *Claassenia* sp. 2. Genes outside the map are transcribed clockwise, whereas genes inside the map are transcribed counterclockwise. The interior circles show GC content and the GC skew, and these are plotted as the deviation from the average value of the entire sequence.

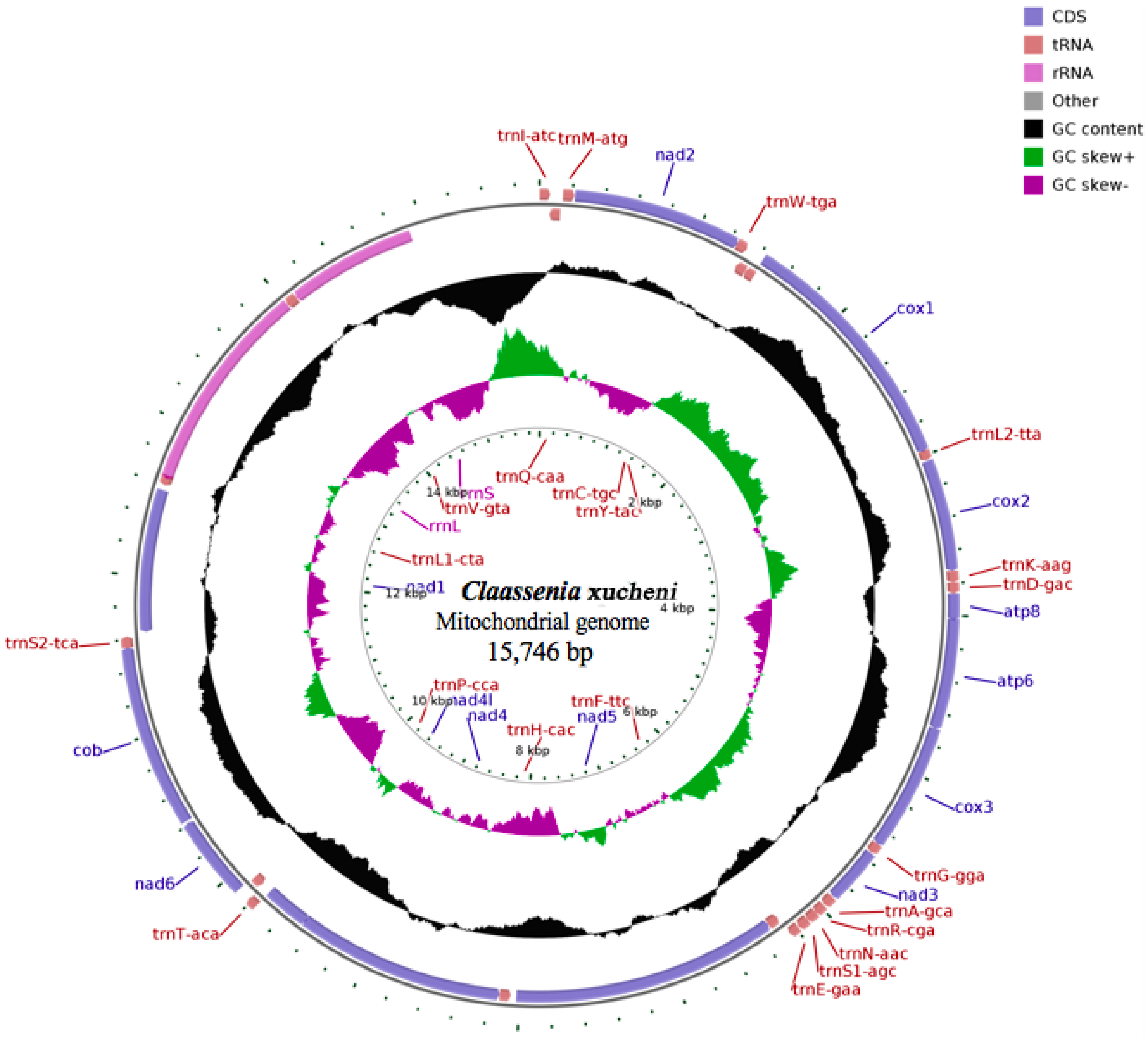

Figure S2. Mitochondrial maps of *C. xucheni*. Genes outside the map are transcribed clockwise, whereas genes inside the map are transcribed counterclockwise. The interior circles show GC content and the GC skew, and these are plotted as the deviation from the average value of the entire sequence.

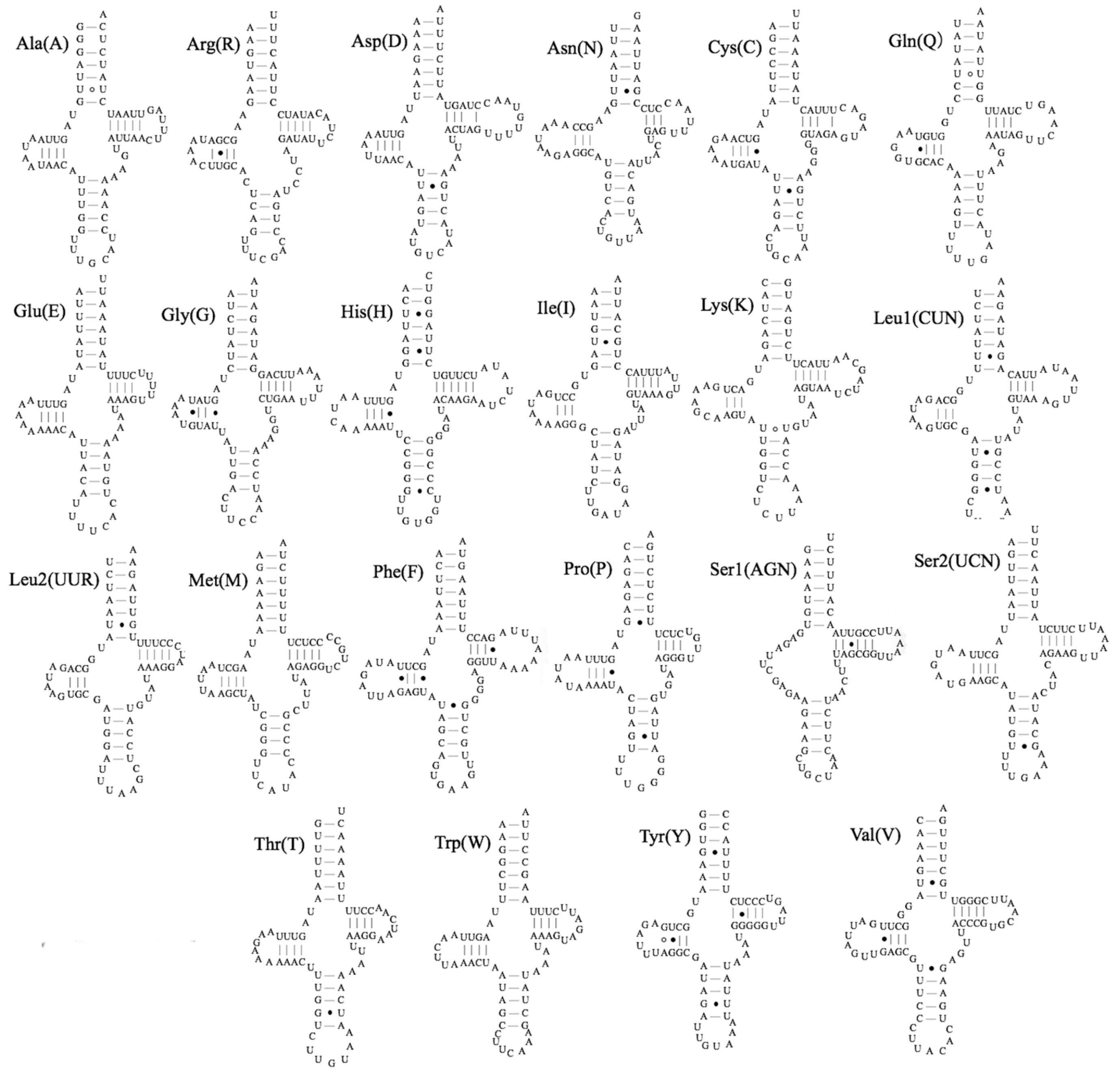

Claassenia sp.2

Figure S3. Predicted secondary structures of tRNAs in *claassenia* sp.2. The tRNAs are labelled with abbreviations of their corresponding amino acids.

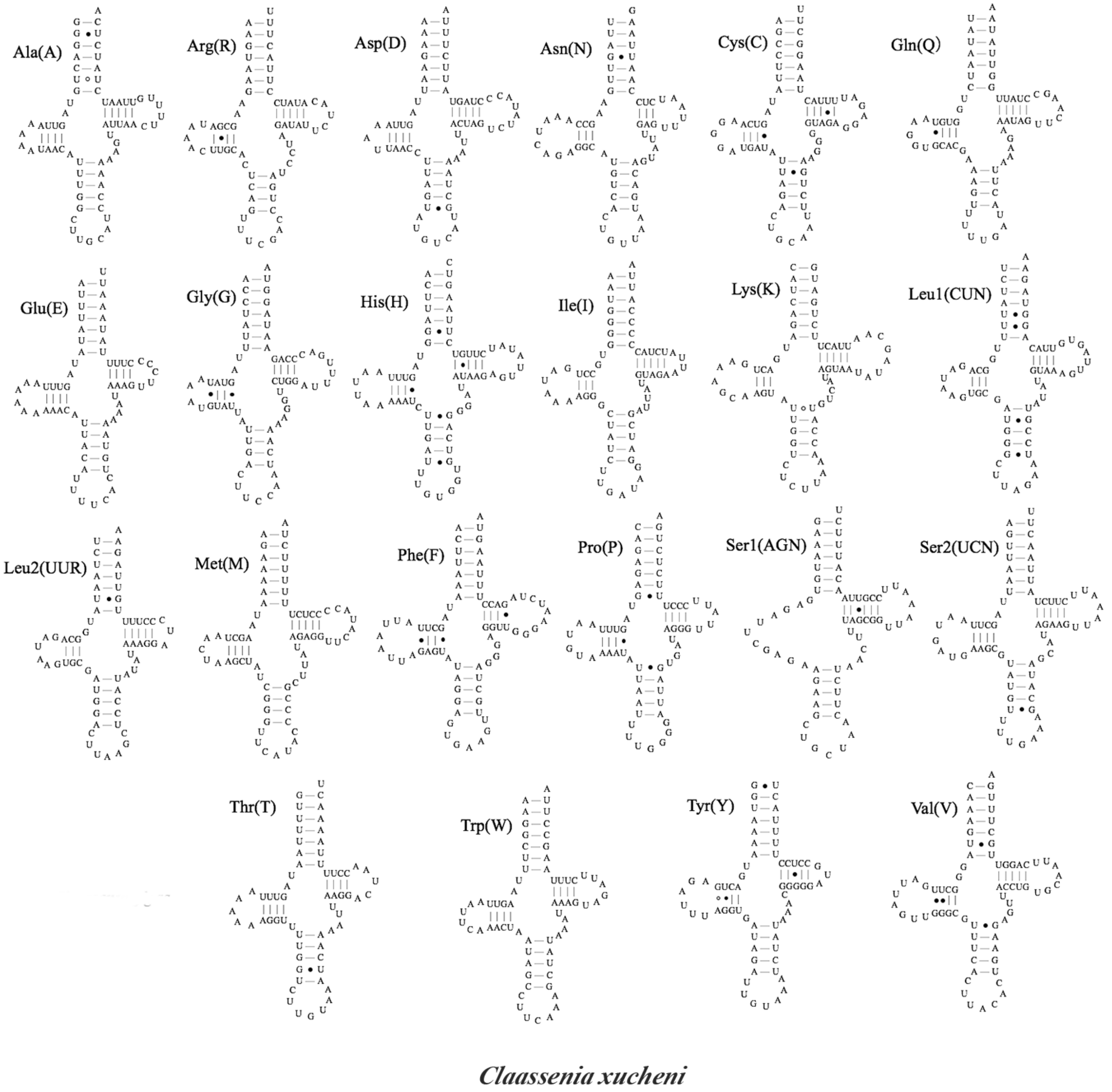

Figure S4. Predicted secondary structures of tRNAs in in *C. xucheni*. The tRNAs are labelled with abbreviations of their corresponding amino acids.
